# The Impact of Country Self-Citation Rate Among Medical Specialties in Saudi Arabia

**DOI:** 10.7759/cureus.12487

**Published:** 2021-01-04

**Authors:** Anas M Bardeesi, Aimun A Jamjoom, Abdulhadi Algahtani, Abdulhakim Jamjoom

**Affiliations:** 1 Department of Surgery, King Saud Bin Abdulaziz University for Health Sciences College of Medicine, Jeddah, SAU; 2 Department of Clinical Neuroscience, Royal Infirmary of Edinburgh, Edinburgh, GBR

**Keywords:** total cites, saudi arabia, self-citation rate, country self-citation, medical specialty, citation metrics, total documents, scimago journal & country rank, bibliometrics, cites per document

## Abstract

Objectives

The purpose of the study was to evaluate the impact of country self-citation rate (SCR) among medical specialties in Saudi Arabia, and to assess the impact of self-citations on the country’s total cites world ranking in different specialties.

Methods

SCImago Journal & Country Rank (SJR) was used to collect data related to all medical specialties in Saudi Arabia for the period 1996-2019. The country SCR for the specialties was correlated with several bibliometric parameters and examined statistically. The specialties that showed a drop in Saudi Arabia’s total cites world ranking following the exclusion of self-citations were identified.

Results

The median country SCR for 46 specialties in Saudi Arabia was 9.5% (range: 4.6-23.1%). The two specialties with the highest country SCR were Public Health (23.1%) and Family Practice (22.9%). Country SCR was significantly higher in the non-clinical specialties compared to clinical specialties (15.3% vs. 9.6%). It did not correlate significantly with any of the examined productivity indices. The exclusion of self-citations resulted in a drop in Saudi Arabia’s total cites world ranking in six (13%) specialties only. There was no significant difference between the country’s total cites and net total cites world rankings in the specialties.

Conclusions

Self-citation may be appropriate and signify an expansion of the authors' previous work. Country SCR in medical specialties in Saudi Arabia is relatively low and not affected by total documents and total cites. Non-clinical specialties tend to self-cite more. The exclusion of self-citations had minimal effect on Saudi Arabia’s total cites world ranking, indicating that country SCR in the specialties is unlikely to impact its international scientific standing. Our findings do not support the argument for eliminating self-citation from citation-based metrics. We believe that more collaborative and global research practices should be encouraged.

## Introduction

It is widely accepted that citation-based bibliometrics can influence careers, funding, and the reputation of researchers and their institutions [1,2]. Citing one’s own work (self-citation) could be a fundamental part of scientific communication that indicates the progressive nature of the research. However, it is often viewed negatively and considered to be a potential gaming tactic that could distort the scientific impact metrics [1]. Self-citation may be categorized according to who does the citing of whom and where, the beneficiaries of the self-citation, the nature of the citing unit, and its appropriateness [2]. It is considered inappropriate when it is unjustified or incorrect as it can replicate flawed beliefs and introduce biases in the citation indices [2,3].

Self-citation rate (SCR) can be measured for authors, journals, specialties, institutions, and countries. The literature on self-citation has been growing in recent years; however, it remains primarily author- and journal-focused [4,5]. A discrepancy in the SCR among the various scientific specialties and a higher SCR in multidisciplinary fields have been reported by some studies [6,7].

A recent review of the self-citation literature has indicated that little attention has been paid to country SCR compared to author and journal SCR [8]. Country self-citation is usually the result of local/national collaboration and researchers' awareness about their compatriots' work and their choosing to cite it. It is typically high as it reflects the summation of self-citations by the individual author and groups of authors as well as institutions from the same country. A considerable part of country SCR, however, is attributed to authors citing their own earlier work [9]. 

SCImago Journal & Country Rank (SJR) [10] is a portal that uses the Scopus database and provides free information relating to the performance of countries in a variety of scientific fields. The data includes several bibliometric indicators such as the total number of documents, citable documents, total cites, self-citations, and cites per document. The site offers countries' productivity data and worldwide ranking relating to 48 medical specialties during 1996-2019. At present, the literature lacks studies that assess the differences in the magnitude of country SCR among various medical specialties within the same country. This prompted us to undertake the current study.

The purpose of the study was to assess the scale of country SCR among various medical specialties in Saudi Arabia, to identify the specialties with relatively high SCR, to correlate the country SCR with several productivity indices, and to determine the impact of excluding self-citations on Saudi Arabia’s total cites worldwide ranking in each of the medical specialties.

## Materials and methods

This study was carried out at the King Khalid National Guards Hospital, Jeddah, Saudi Arabia. No ethical approval was needed for the study since the study was based on information obtained from open-access sources. The SJR site [10] was searched on November 1, 2020, using the following items: subject area “medicine”, country “all regions”, year “1996-2019”, and subject category “each of the listed 48 specialties in the SJR site”. The subject categories “drug guides” and “medical reviews and references” were excluded due to limited contributions to the same by researchers from Saudi Arabia.

Using various SJR country ranking tables, the following data were collected for each of the 46 medical specialties in Saudi Arabia for the period 1996-2019: total citable documents, total cites, total self-cites, total citable documents, and total cites world rankings. The country SCR for each specialty was calculated by dividing the total self-cites by the total cites and expressed as a percentage. The cites per document were calculated by dividing the total cites over the total citable documents. The net total cites were calculated by subtracting the total self-cites from the total cites. Saudi Arabia’s total cites world ranking in each of the 46 specialties was revised using the net total cites score. The 46 medical specialties were categorized as clinical and non-clinical. The 13 non-clinical specialties were as follows: Anatomy, Biochemistry, Embryology, Epidemiology, Health Informatics, Health Policy, Histology, Immunology, Microbiology, Pathology, Pharmacology, Physiology, and Public Health. The remaining 33 specialties were considered clinical. The country SCR for clinical and non-clinical specialties was compared by calculating the mean difference (MD) and 95% confidence interval (CI) between the two groups. The MedCalc statistical software [11] was used to calculate the MD, and a p-value of less than 0.05 was considered statistically significant.

The country SCR findings for specialties in Saudi Arabia were correlated with the total citable documents, total cites, cites per document, the total cites world rankings, and net total cites world rankings. Spearman's Rho coefficient test was used for the analysis. The impact of excluding self-citations was weighed by determining the difference between Saudi Arabia’s total cites and the net total cites world rankings in every specialty. The extent of the variation between the country’s total cites and net total cites country rankings was determined by correlating the two rankings by using the Wilcoxon signed-rank test. The correlation analyses were performed using Social Science Statistics [12], and a p-value of less than 0.05 was considered statistically significant.

## Results

The country SCR and other productivity parameters for 46 medical specialties in Saudi Arabia during 1996-2019 are summarized in Table 1.

**Table 1:** The country SCR and other productivity parameters for 46 medical specialties in Saudi Arabia during 1996-2019 (ranked by SCR) SCR: self-citation rate; Doc.: document; Cit.: citable; Med.: Medicine; Obs.: Obstetrics; Misc: miscellaneous

Medical Specialty	SCR (%)	Total Cit. Doc. (a)	Total Cites (b)	Total Self-Cites (c)	Net Cites (b-c)	Cites per Doc (b/a)	Total Cit. Doc. Rank	Total Cites World Rank	Net Cites World Rank	Net Total Cites Rank Change
Public Health	23.1	2,572	25,622	5,923	19,699	10	47	54	54	0
Family Practice	22.9	60	275	63	212	4.6	38	51	53	2
Health Policy	20.8	494	5,012	1,041	3,971	10.2	48	47	46	-1
Epidemiology	20.3	703	12,370	2,514	9,856	17.6	39	44	46	2
Infectious Disease	20.1	2,858	45,663	9,174	36,489	16	41	48	49	1
Health Informatics	19.8	1,151	6,412	1,270	5,142	5.57	31	34	34	0
Microbiology	19.2	1,178	22,555	4,335	18,220	19.2	40	42	42	0
Complementary Med.	16.1	789	9,140	1,472	7,668	11.6	27	31	31	0
Biochemistry	15.2	596	9,184	1,393	7,791	15.4	33	37	37	0
Anatomy	14.4	382	3,149	452	2,697	8.2	37	42	41	-1
Medicine (Misc.)	13.9	29,140	413,477	57,593	355,884	14.2	39	40	40	0
Pharmacology	13.8	1,762	17,119	2,357	14,762	9.7	36	42	42	0
Embryology	13.1	58	725	95	630	12.5	42	44	44	0
Endocrinology	12.5	1,368	21,663	2,718	18,945	15.8	39	40	39	-1
Histology	12.5	469	4,935	616	4,319	10.5	40	41	41	0
Psychiatry	12.2	1,021	8,849	1,077	7,772	8.7	43	53	52	-1
Pediatrics	11.1	2,277	20,429	2,272	18,157	9	38	38	38	0
Gastroenterology	10.1	750	11,513	1,165	10,348	15.4	41	43	43	0
Genetics	10.8	1,141	25,624	2,762	22,862	22.5	33	38	38	0
Pulmonology	10.5	970	15,702	1,656	14,046	16.1	37	39	40	1
Oncology	10.3	2,269	34,010	3,487	30,523	15	38	42	42	0
Pathology	10	1,099	13,551	1,354	12,197	12.3	40	40	38	-2
Geriatrics	9.6	179	2,377	229	2,148	13.3	43	44	43	-1
Radiology	9.4	1,838	20,207	1,892	18,315	11	36	38	38	0
Ophthalmology	9.3	1,914	26,199	2,431	23,768	13.7	25	27	27	0
Dermatology	9.2	766	8,147	748	7,399	10.6	37	40	41	1
Internal Medicine	9	601	10,576	953	9,623	17.6	40	41	41	0
Transplantation	8.8	579	7,670	678	6,992	13.3	32	36	36	0
Cardiology	8.7	2,270	31,251	2,710	28,541	13.8	39	44	48	4
Immunology	8.7	1,025	19,477	1,692	17,785	19	41	46	46	0
Hematology	8.5	1,048	16,450	1,399	15,051	15.7	40	41	41	0
Surgery	8.5	3,412	34,173	2,912	31,261	10	35	39	38	-1
Physiology	8.3	481	10,373	857	9,516	21.6	44	41	39	-2
Rehabilitation	8.3	281	2,358	196	2,162	8.4	41	45	45	0
Neurology	8.2	2,122	25,225	2,061	23,164	11.9	38	40	40	0
Orthopedics	8	682	8,343	668	7,675	12.2	44	45	44	-1
Emergency Medicine	7.8	293	2,965	230	2,735	10.1	32	35	35	0
Nephrology	7.4	410	5,661	419	5,242	13.8	39	42	42	0
Otorhinolaryngology	7.2	902	7,774	559	7,215	8.6	30	36	36	0
Rheumatology	6.6	348	5,913	393	5,520	17	45	47	47	0
Obs. and Gynecology	6.4	983	14,268	907	13,361	14.5	44	42	42	0
Reproductive Med.	5.5	437	6,852	375	6,477	15.7	40	41	41	0
Urology	5	754	8,516	430	8,086	11.3	33	38	38	0
Hepatology	4.7	260	7,897	370	7,527	30.4	42	43	43	0
Critical Care Medicine	4.6	452	14,150	647	13,503	31.3	37	37	37	0
Anesthesiology	4.6	771	8,416	386	8,030	10.9	35	37	36	-1
Median	9.5	780	10,475	1,121	9,570	13.3	39	41	41	0
Range	4.6-23.1	58-29,140	275-413,477	63-57,593	212-355,884	4.6-31.3	25-47	27-54	27-54	(-2)-(+4)

The median (range) results for the specialties were as follows: country SCR: 9.5% (4.6-23.1%), total citable documents: 780 (58-29,140), total cites: 10,475 (275-413,477), total self-cites: 1,121 (63-57,593), net total cites: 9,570 (212-355,884), cites per document: 13.3 (4.6-31.3), total citable documents world ranking: 39 (25-47), total cites world ranking: 41 (27-54), net total cites world ranking: 41 (27-54), and net total cites rank change: 0 [(-2)-(+4)].

The 10 medical specialties in Saudi Arabia that had the highest country SCR during the study period (and their SCR score) were as follows: Public Health (23.1%), Family Practice (22.9%), Health Policy (20.8%), Epidemiology (20.3%), Infectious Disease (20.1%), Health Informatics (19.8%), Microbiology (19.2%), Complementary Medicine (16.1%), Biochemistry (15.2%), and Anatomy (14.4%). The median findings for these specialties were as follows: country SCR: 20%, total citable documents: 746, total cites: 9,162, total self-cites: 1,433, net total cites: 7,730, cites per document: 10.9, total citable documents world ranking: 38.5, total cites world ranking: 43, net total cites world ranking: 44, and net total cites rank change: 0. The mean [±standard deviation (±SD)] country SCR for the 13 non-clinical specialties was 15.3% (±4.9%) and for the 33 clinical specialties was 9.6% (±4%). The difference between them was significant [MD (95% CI)=5.7% (2.89-8.51%)] (p=0.0002).

The comparative analysis between the country SCR for the 46 medical specialties in Saudi Arabia and the bibliometric parameters showed no significant correlation between the country SCR and total citable documents (Rs=0.2499) (p=0.0938), total cites (Rs=0.1295) (p=0.3909), cites per document (Rs = -0.2122) (p=0.1569), total citable documents world ranking (Rs=0.0169) (p=0.9115), and total cites world ranking (Rs=0.2249) (p=0.1329).

The impact of excluding self-citations on Saudi Arabia’s total cites world ranking in the 46 specialties is shown in Table 1. It had no effect on the ranking of as many as 30 (65%) specialties. Furthermore, 10 (22%) specialties improved their ranking, with a one rank-gain in eight specialties (Health Policy, Anatomy, Endocrinology, Psychiatry, Geriatrics, Surgery, Orthopedics, and Anesthesiology) and two-rank gain in two specialties (Pathology and Physiology). However, the exclusion of self-citations diminished Saudi Arabia’s total cites world ranking in six (13%) specialties, leading to a one-rank drop in three specialties (Infectious Disease, Pulmonology, and Dermatology), a two-rank drop in two specialties (Epidemiology and Family Practice), and a four-rank drop in one specialty (Cardiology). The median results for the six specialties whose rankings were impacted by the exclusion of self-citations were as follows: country SCR: 15.3%, total citable documents: 868, total cites: 14,036, total self-cites: 2,085, net total cites: 11,951, cites per document: 14.9, total citable documents world ranking: 38.5, total cites world ranking: 44, net total cites world ranking: 47, and net total cites rank change: +1.5. In addition, there was no significant difference in the correlation between Saudi Arabia’s total cites and net total cites world rankings (Z = -0.362) (p=0.7188).

## Discussion

Authors may choose to cite their own papers to make their earlier work noticeable, to highlight an argument, or to project an image of scientific expertise [13,14]. The median country SCR for 46 specialties in Saudi Arabia was 9.5%, which is much lower than the documented country SCR range of 17.8-54.9% for the 10 most productive countries in the world [8]. The relatively low SCR among medical specialties in Saudi Arabia is not surprising as it is generally accepted that the nations with a greater share of world publications are more likely to self-cite [8]. Additionally, the discrepancy in the rates between various reports could be attributed to the differences in duration of the study period, the specialties covered, the data sources, and the inclusion criteria. Author and journal SCR are reported to have lower rates (range: 2.2-10%) [4,5,15,16] and have been on the decline in recent years [17,18]. On the other hand, country SCR has been on the rise in many nations [8,19]. Recent reports state that the referencing system among the major science-producing countries remains heavily oriented towards national papers in biomedical sciences [9]. It is also reported that the citation national bias was higher for scientific fields of greater national interest than the ones that are more universal [20]. China, the USA, and Iran are recognized to have high country SCR among nations [19]. Iran specifically has a disproportionately high SCR, being ranked third in the world based on SCR and 22nd based on total productivity [21].

The 10 specialties that had the highest country SCR in Saudi Arabia during 1996-2019 were Public Health, Family Practice, Health Policy, Epidemiology, Infectious Disease, Health Informatics, Microbiology, Complementary Medicine, Biochemistry, and Anatomy. Their SCR ranged from 14.4% to 23.1%. As expected, their median country SCR was considerably higher than that of the 46 specialties (20% vs. 9.5%). However, they had a slightly lower median total citable documents (746 vs. 780), total cites (9,162 vs. 10,475), and cites per document (10.9 vs. 13.3). This is not surprising as in this study the country SCR for the 46 specialties did not correlate significantly with total citable documents, total cites, and cites per document. However, in the literature, it is clearly documented that self-citation correlates positively with total publications [8,15], total cites [8,13,16], and the number of authors [3,4,6,13,16]. Our analysis showed that non-clinical specialties had a significantly higher country SCR compared to clinical specialties (15.3% vs. 9.6%). In the literature, a higher SCR is found in basic sciences compared to clinical research [20], in specialty journals compared to general ones [3,5,18], in original articles compared to reviews and case reports [16], and in studies with smaller sample size [4].

It is recognized that the exclusion of self-citations is less likely to affect the total cites world ranking for the highly cited, highly-ranking countries [8]. The elimination of self-citations had no impact on the country total cites ranking of 65% of the specialties and led to an improvement in the ranking in 22% of the specialties in Saudi Arabia. It impacted the ranking negatively for six (13%) specialties only. The affected specialties were Family Practice, Epidemiology, Infectious Disease, Pulmonology, Dermatology, and Cardiology. They had a median country SCR that was higher than that for the 46 specialties (15.3% vs. 9.5%). However, they had a lower median total citable documents (868 vs. 780), but a higher median total cites (14,036 vs. 10,475) and cites per document (14.9 vs. 13.3). It is known that self-citation occurs more in multidisciplinary publications [7] and that certain specialties such as Psychology, Cardiology, and Infectious Disease are associated with higher SCR [4,7]. The variation in productivity and worldwide ranking among medical specialties in Saudi Arabia has been highlighted [22]. Fourteen out of the 46 medical specialties were identified as having a positive relative contribution to the literature, implying that the country had a higher share in the world publications in that specialty compared to its overall share in the world total publications [22]. Saudi Arabia’s total cites world ranking was negatively impacted by the exclusion of self-citations in only three of the previously identified 14 specialties with a positive contribution [22]. These specialties were Epidemiology, Infectious Disease, and Pulmonology. The relatively low country SCR for specialties in Saudi Arabia and the fact that there was no significant difference between the country’s total cites and the net total cites world rankings indicate that self-citation among medical specialties in Saudi Arabia has minimal impact on its total cites world ranking. Hence, our findings do not support the argument for eliminating or modifying the use of self-citation in the citation-based metrics, which were suggested by others [3,5]. 

In the literature, it has been shown that broadening the network through international collaboration can influence country SCR significantly [8,9,19]. Baccini et al. [23] studied the self-citation among G10 countries during 2000-2016 and identified Italy as the country with the highest level of inwardness and the lowest rate of international collaboration. They attributed that to the strategic use of citations in the Italian scientific community. Country SCR is also higher among nations facing language barriers [15]. Yang et al. [24] found that Chinese journals have a significantly higher SCR than non-Chinese international journals and ascribed that to Chinese journals having lower visibility and being more isolated. Yaminfirooz et al. [21] have highlighted the need for researchers to consider other modes of scientific visibility to decrease the country SCR in Iran.

There are some limitations to this study. The study was heavily dependent on the accuracy of the search engine SJR and the exactness of the allocation of data under various specialties. It is possible that there were imprecisions, particularly with respect to specialty and topic overlap. The contribution of Saudi Arabia’s researchers to multi-national articles could not be defined. The influence of having a Saudi Arabia specialty journal on the country SCR for the specialties was not examined due to the limited number of Journals from Saudi Arabia on the SJR website.

## Conclusions

Self-citation is not necessarily inappropriate by default. It may be needed at times and may reflect an expansion on earlier research. Country SCR in medical specialties in Saudi Arabia is relatively low (median: 9.5%), and it is not influenced by total documents and total cites. Non-clinical specialties in Saudi Arabia are more likely to self-cite. The two specialties with the highest SCR among the 46 specialties in Saudi Arabia during the period were Public Health (23.1%) and Family Practice (22.9%). The exclusion of self-citations had minimal effect on Saudi Arabia’s total cites world ranking. Our findings imply that country SCR among the 46 specialties is unlikely to change Saudi Arabia’s international scientific standing. The results also do not support the argument for eliminating self-citations from citation-based metrics. We recommend that more collaborative and global research practices be encouraged.
